# Effect of Magnetic Field Treatment at Different Intensities on the Quality and Biological Properties of Bread Produced with *Saccharomyces cerevisiae*

**DOI:** 10.1021/acsomega.6c01691

**Published:** 2026-06-22

**Authors:** Gökhan Akarca, Azize Atik, İlker Atik, Ayşe Janseli Denizkara, Yunus Çetintaş

**Affiliations:** † Faculty of Engineering, Department of Food Engineering, 53002 Afyon Kocatepe University, Afyonkarahisar 03200, Turkey; ‡ Afyon Vocational School, Food Technology Program, Afyon Kocatepe University, Afyonkarahisar 03200, Turkey; § Ula Ali Kocman Vocational School, Food Processing Department, Food Quality Control and Analysis Program, 52986 Mugla Sitki Kocman University, Muğla 48640, Turkey; ∥ Central Research Laboratories, Food Analysis Application and Research Center, Mugla Sitki Kocman University, Muğla 48000, Turkey

## Abstract

This study investigated the effects of magnetic field exposure at different intensities (180 μT, 240 μT, and 300 μT) on the fermentation efficiency, physicochemical properties, and biological characteristics of bread produced using *Saccharomyces cerevisiae*. The magnetic field greatly affected yeast metabolism. Bread pH and *a*_w_ values were reduced after treatment. Isolated yeast samples under 240 μT magnetic field intensity showed the lowest pH and *a*_w_ values of 4.35 and 0.963, respectively. All dough samples prepared with magnetic field-treated cultures had lower TPA values. Bread samples made from these doughs had higher leavening rates, pore diameter, and TPA values. The most substantial drop in dough samples and increase in bread samples were observed in cultures exposed to a 240 μT magnetic field strength. The highest quantities of lactic, ascorbic, oxalic, tartaric, and malic acids in bread samples were found in cultures exposed to 240 μT magnetic field intensity (291 mg/kg, 0.817 mg/kg, 0.296 mg/kg, 0.110 mg/kg, and 3.955 mg/kg). Sourdough culture samples showed more alterations than commercial culture samples. Yeasts exposed to magnetic field application synthesized higher amounts of CO_2_ and metabolites, such as organic acids, during fermentation, improving dough and bread texture and nutritional value. This study suggests that magnetic field treatment may enhance certain techno-functional and nutritional characteristics of bread.

## Graphical abstract

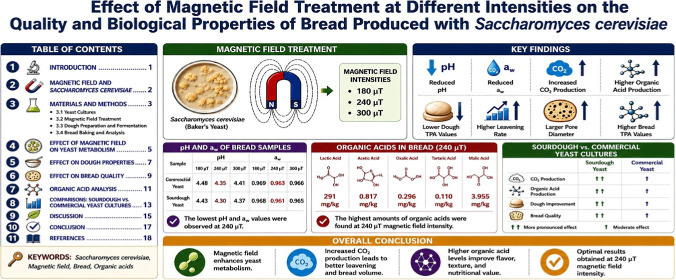

## Introduction

Bread, a staple food consumed worldwide, is a complex carbohydrate-rich product whose nutritional properties can be influenced by various factors. Starch, protein, lipids and other compounds found in wheat flour play a critical role in determining the quality and nutritional value of bread.^1^ The bread-making procedure can influence the nutritional characteristics of bread. Fermentation, leavening, and baking can impact the availability and bioavailability of specific nutrients. Moreover, fortifying wheat flour or the final bread product can enhance its nutritional content by incorporating micronutrients or other beneficial chemicals.^2^

*Saccharomyces cerevisiae*, or baker’s yeast, is a significant microorganism used in producing many fermented foods and beverages, such as bread. This unicellular eukaryotic fungus is extensively utilized in the baking industry for its capacity to convert carbohydrates into carbon dioxide and ethanol via fermentation.^3,4^ The fermentation conducted by *S. cerevisiae* is fundamental to bread making. In this process, yeast cells metabolize available carbohydrates, primarily glucose and sucrose, to produce carbon dioxide and ethanol as byproducts. The carbon dioxide produced by the yeast induces the dough to leaven, resulting in the airy, light texture characteristic of well-fermented breads.

The fermentation process not only provides leavening but also enhances the flavor characteristics of the bread. The synthesis of ethanol and various volatile compounds, including aldehydes, ketones, and alcohols, can contribute a spectrum of intricate tastes to the final bread product.^3^ *S. cerevisiae* exhibits a remarkable capacity to adapt to various environmental stresses, including severe temperatures, oxidative stress, osmotic fluctuations, and pH changes.

The stress response mechanisms involve a complex interaction of cellular activities, including the synthesis of compatible osmolytes, the dynamic restructuring of the cell wall, and a temporary interruption of the cell division cycle. All of these collectively contribute to the impressive cellular survival capabilities of this model eukaryotic organism under adverse conditions. In particular, the heat shock response pathway in *S. cerevisiae* has been extensively characterized and has served as the primary model system to explain the complex regulation and important functions of heat shock proteins in the broader context of eukaryotic cell biology.^5^

Innovative technologies are often employed to enhance food production processes and improve the quality of the end product. Magnetic fields have emerged as a versatile tool in the food production industry, offering various applications and potential benefits. These fields can be static, oscillating, or pulsed, and their effect on food properties is determined by factors such as flux density, frequency, polarity, and exposure time.^6^ Although the stress responses of *S. cerevisiae* have been thoroughly investigated under diverse environmental stressors, the effects of magnetic field exposure on the metabolic and physiological adaptations of this yeast species remain a comparatively under-explored domain. Recent research suggests that exposure to magnetic fields may elicit various cellular responses in *S. cerevisiae*, potentially including alterations in gene expression patterns, enzyme activity, biomass, and the synthesis of industrially relevant metabolites.^4,5,7–10^

These responses include changes in gene expression patterns,^4^ modulation of enzyme activities and changes in biomass production and industrially relevant metabolites.^5^ For example, research has demonstrated that when *S. cerevisiae* is exposed to magnetic fields, it upregulates genes related to stress response, such as heat shock proteins. This suggests that the microorganism may activate adaptive mechanisms to deal with this physical stimulation.^5^ Applying a magnetic field influences the activities of key metabolic enzymes, including those involved in glycolysis and respiration, subsequently impacting the overall cellular energy metabolism and growth dynamics of this yeast species.^4^

Despite increasing interest in electromagnetic field applications in food biotechnology, the effects of magnetic field exposure on the fermentation performance of *S. cerevisiae* during bread production remain insufficiently characterized.

This study aimed to evaluate the effects of magnetic field exposure on fermentation behavior, physicochemical properties, textural characteristics, and organic acid composition of bread produced with commercial and isolated *S. cerevisiae* strains.

## Methodology

### Materials

All ingredients used for bread making, including wheat flour (*Triticum aestivum*), iodine-free salt, instant yeast, sunflower oil, skim milk powder, and sugar, were purchased from a local market in Afyonkarahisar, Türkiye.

### Instant Yeast

Instant yeast (Dr. Oetker, Turkey), containing *S. cerevisiae* strains, was used to make the bread.

### Isolated Yeast Strains

The isolated *S. cerevisiae* strains used in bread making were isolated from traditional bread made in Afyonkarahisar province within the scope of another study on sourdough. The isolation and verification of *S. cerevisiae* strains consisted of culture and molecular identification techniques and biochemical tests.

### System Design

The system design was developed explicitly for this purpose, utilizing aluminum material (Figure 1). The device’s rotating part is movable with the help of a rotor. Inside, there is a fixed part where the culture samples are placed. The rotating part is shaped like a square prism, contains 12 interchangeable neodymium type magnets (3 on each side) and is connected to a rotor with adjustable rotation speed. The distance between the magnets and the samples was 5 cm.

**Figure 1:**
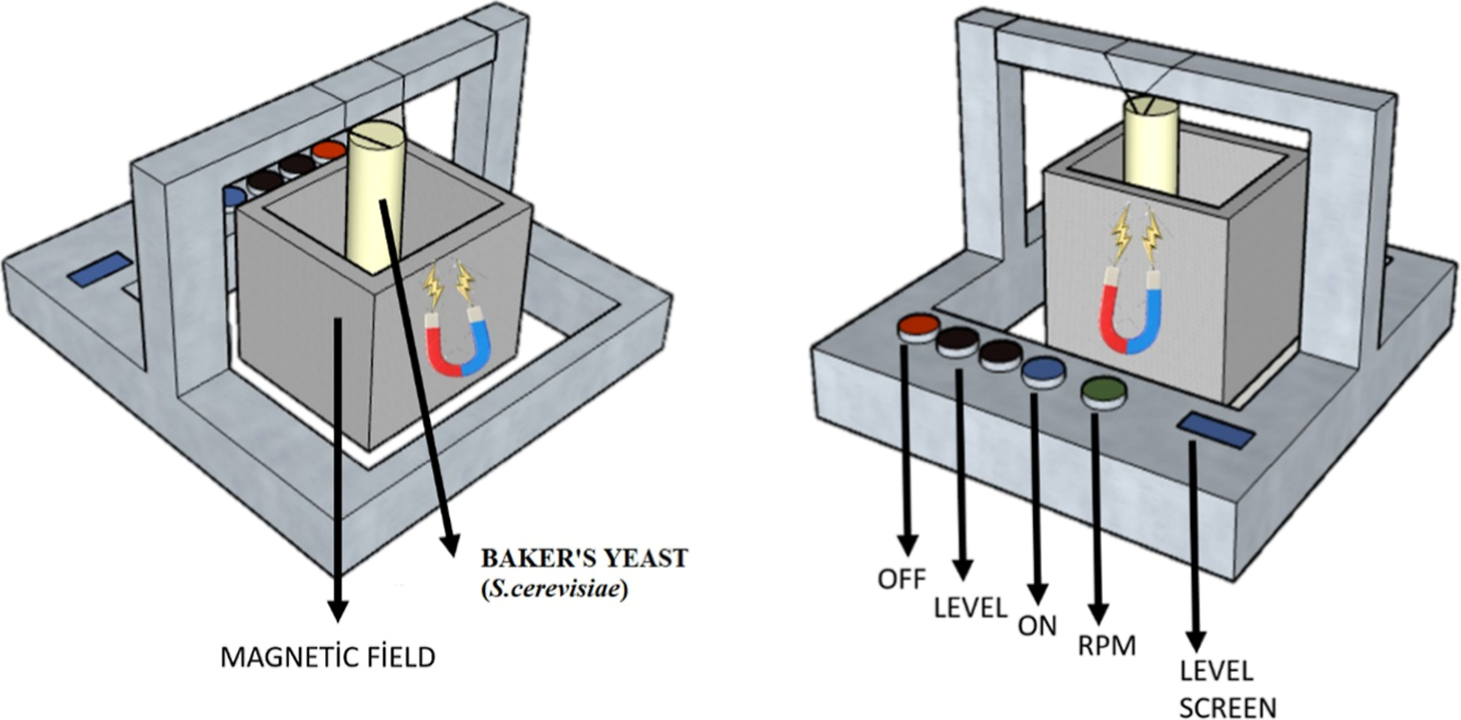
Magnetic field design.

The rotor speed was maintained at a constant rate of 80 rpm during the investigation. Following each experiment, the magnets were replaced, resulting in magnetic fields of three distinct strengths. The strength of each magnet was quantified as 15, 20, and 25 μT. Accordingly, magnetic fields of 180, 240, and 300 μT were generated in the applications. The strength of the magnetic field generated during the research was measured continuously with the help of a tesla meter (3B Scientific, Germany). The magnetic field strength was measured in the range of 183–196 μT for Trial 1, 236–264 μT for Trial 2 and 296–311 μT for Trial 3.

### Strains and Their Preparation for Inoculation

Commercial and isolated *S. cerevisiae* strains used in the study were incubated in YPD (yeast extract, peptone, dextrose) broth at 25 °C for 48 h. The cultures were propagated in 5 L of YPD broth to obtain sufficient biomass prior to inoculation. After the incubation periods, the strains were subjected to centrifugation at 7000 rpm for 10 min at 22 °C, and the resultant pellets were washed twice with PBS (phosphate buffered saline, Merck, 524650, Germany). The obtained pellets were sampled and standardized to a turbidity of 0.5 McFarland (10^7^ to 10^8^ cfu/g) utilizing a densitometer (Biosan 1B, Turkey). Then, 1 mL of this mixture was added to the bread mixture. The purity of the inoculated mixtures was verified using microbiological investigation.^11^ It was confirmed through microbiological analyses that the yeast counts inoculated into the bread mixture are in the range of 10^7^ to 10^8^ log cfu/mL.

### Making of the Bread Samples

The Tefal (PF2101, Paindore, France) bread-making machine was used to make the bread samples used in the study. The formulation recommended by the device manufacturer was used in the production. The formulation was prepared as follows: 82.5 mL of water (at 37 °C), 7 mL of sunflower oil, 1.4 g of salt, 4.25 g of beet sugar, 8.75 g of skim milk powder, 150 g of flour, and yeast (7–8 log cfu/g) for 250 g of bread. All ingredients were mixed in the device chamber for 10 min. Then, the dough inside was removed from the device and placed in the chamber of the specially designed device. The rotor was started, and the magnets were rotated around the sample for 60 min. The bread dough was then removed from the device chamber, placed in an incubator (Incucell, MMM, Germany) and left to ferment at 30 °C for 60 min. During fermentation, pH values of all samples were measured every 10 min, and pH changes were recorded. At the end of fermentation, the chamber and its contents were returned to the device, which was then operated in the roll bread program and baked at 200 °C for 50 min.

### Physicochemical Analyses

A 10 g bread sample was mixed with 10 mL of distilled water and homogenized using a homogenizer (Daihan WiseStir, HS-30T, South Korea). The pH values of the prepared mixtures were measured using a pH meter (HANNA, HI 2215 pH/ORP meter) according to the AOAC 981.12 method. The dry matter content of the samples was determined according to AOAC 925.09 standards.^12,13^ Water activity values of the samples were determined according to the AOAC 978.18 method using a water activity analyzer (Novasina LabTouch-aw, Lachen, Switzerland).^14^ The volume of the bread samples was measured using the mustard seed displacement method, and the specific volume was calculated by dividing the volume values (cm^3^) by the weight of the bread (g).^15^ Dough analyses were conducted postfermentation, whereas bread analyses were executed following the baking and subsequent cooling of the bread at room temperature for 6 h.

### Leavening Ratio and Pore Diameter

The leavening ratio and pore diameters of the samples were quantified with a digital caliper after the bread had cooled to room temperature for 6 h postbaking. The leavening ratio values of the samples were determined using modified methodologies established by refs 15 and 16 .

The pore diameter analysis method described by ref 9 was revised. By this approach, uniform slices of bread were extracted from each loaf. The dimensions of five pores, excluding cracks and holes, situated in the center of each slice, were measured and computed to obtain average values.

### Texture Profile Analysis (TPA)

The texture profile analysis (TPA) of the bread samples was conducted at 22 °C with a texture analyzer (TA.TX, Stable Micro Systems, Godalming, Surrey, UK). The samples were cut into 20–25 mm-thick slices, and the qualities of hardness, cohesiveness, adhesiveness, elasticity, and chewiness were assessed. A cylindrical probe with a diameter of 12.5 mm and a load cell weighing 5 kg was utilized in the analysis. The probe velocity before the TPA test was established at 2 mm/s, the testing velocity at 3 mm/s, the post-test velocity at 3 mm/s, the dwell duration at 5 s, the trigger force at 10 g, and the deformation rate at 50%.^17^

### Organic Acid Analysis

The extraction of organic acids from bread was performed according to the modified method of.^18^ Briefly, 10 g of the sample was added to 100 mL of ultrapure water, and the mixture was homogenized and centrifuged at 8000*g* for 15 min. The upper liquid was filtered through a 0.22 μm membrane filter, diluted with 3 mL of ultrapure water and injected into the HPLC (Agilent 1100 series, USA) system. The injection volume was set at 20 μL.

HPLC analysis was performed with an Agilent 1100 series system equipped with a Spherisorb S5 ODS2 column (5 μm, 4.6 × 250 mm) and a photodiode array detector. The mobile phase consisted of ultrapure water acidified with phosphoric acid at pH 2.1 under isocratic conditions at 25 °C. The column temperature was maintained at 25 °C using a column oven. The flow rate was adjusted to 0.6 mL/min. The wavelength of the detector was set to 210 nm.

The organic acid concentration was determined by a calibration curve constructed with organic acid standards (acetic acid, lactic acid, citric acid and succinic acid) prepared in the range of 10–1000 mg/L. The results were expressed as mg organic acid/kg sample.

### Statistical Analysis

A two-way analysis of variance (ANOVA) was conducted to assess the effects of factors and their interactions on the measured parameters, with significance set at *p* < 0.05. All analyses were performed using two independent replicates, each analyzed in parallel (duplicate determinations). Interactions between applied magnetic field intensity and application time were further evaluated by correlation analysis. Mean comparisons were carried out using Duncan’s multiple range test. Statistical analyses were performed using SPSS (version 28). The experiment followed a completely randomized design with replications.

## Results and Discussion

Figure 2 illustrates the fermentation profiles of the samples over time. The pH values of all samples decreased significantly during fermentation (*p* < 0.05). Statistical analysis showed that samples I2 (180 μT) and I3 (240 μT) experienced a significantly greater pH reduction compared to the control (I1), indicating accelerated yeast fermentation under these magnetic field intensities (*p* < 0.05). In contrast, sample I4 (300 μT) maintained higher pH values than the control throughout fermentation (*p* < 0.05). The highest fermentation rate was observed in sample I2, whereas the slowest occurred in sample I3. These findings suggest that moderate magnetic field intensities enhance fermentation, while higher intensities may inhibit the process.

**Figure 2:**
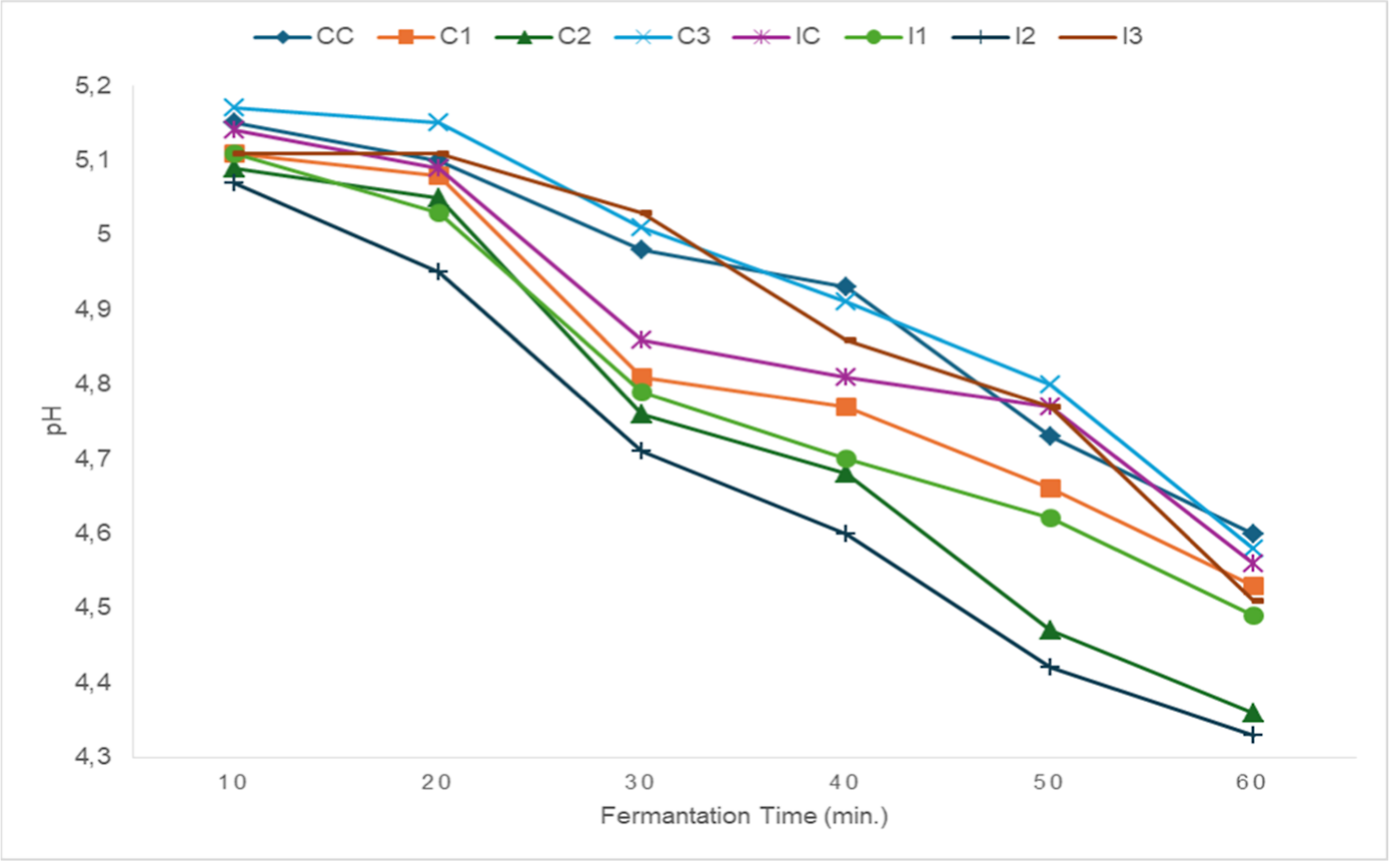
Changes in the fermentation time. CC: commercial control, C1: commercial culture exposed to 180 μT, C2: commercial culture exposed to 240 μT, C3: commercial culture exposed to 300 μT, IC: isolated control, I1: isolated culture exposed to 180 μT, I2: isolated culture exposed to 240 μT, I3: isolated culture exposed to 300 μT.

Low-intensity magnetic fields have been reported to positively influence microbial growth and metabolism, whereas higher intensities or prolonged exposures can inhibit or even halt microbial activity.^19,20^

In line with these findings, our study demonstrated that magnetic field applications of 180–240 μT significantly enhanced fermentation, as evidenced by accelerated pH decline and increased fermentation rates in samples I2 and I3. In contrast, exposure to 300 μT reduced fermentation efficiency, consistent with previous reports that excessive magnetic field strength suppresses microbial activity.^20^

Mechanistically, magnetic fields are thought to enhance cell membrane permeability and modulate ion transport, thereby reducing ATP requirements during biomass synthesis and promoting faster growth and metabolite production.^21^ These effects are consistent with studies on *S. cerevisiae*, where moderate magnetic field exposures (100–250 μT) increased CO_2_ production and ethanol yield during sugar fermentations.^22,23^ Similarly, Sincak et al.^24^ reported that moderate static magnetic field exposures (10–15 mT) significantly increased biomass production and accelerated glucose and oxygen consumption in yeast fermentations, although the effects on metabolite profiles varied with cultivation conditions.

In agreement with these reports, our results indicated that magnetic field applications were more effective in isolated yeast cultures than in commercial mixed cultures, with higher fermentation rates and more pronounced pH decreases in the isolated cultures. At higher field intensities, biomolecular and chemical effects—including altered electronic spin states of reaction intermediates—can disrupt intracellular ion homeostasis, enzyme activities, cellular morphology, growth, and division.,^25–28^ which explains the reduced fermentation efficiency observed in sample I4 (300 μT). Overall, these findings align with previous fermentation studies, demonstrating that moderate magnetic field applications can enhance yeast activity and metabolite production, while excessive exposure may inhibit microbial growth and reduce fermentation efficiency.

The pH and *a*_w_ results of bread samples are given in Table 1. According to the results of the variation analysis, it was determined that yeast strain, magnetic field intensity, and yeast strain × magnetic field intensity interactions were very highly significant (*p* < 0.001) on the pH value. In addition, yeast strain and magnetic field intensity interactions were highly significant (*p* < 0.01) on *a*_w_ values. In the results of the correlation analysis, it was revealed that yeast strain interaction had a very highly negative correlative effect on the pH value and a highly correlative effect on the *a*_w_ value. The pH values of the samples subjected to magnetic field application were lower than those of the control samples (p < 0.05). The commercial culture control sample (CC) had the highest pH value at 4.94, while the I2 sample, isolated from sourdough samples exposed to a magnetic field strength of 240 μT, had the lowest pH value at 4.35. The pH values of the bread samples made with isolated yeasts were lower than those made with commercial culture yeasts. Isolated yeasts exhibited more susceptibility to magnetic field applications than cultured yeasts.

**Table 1:** pH, *a*_w_ and Dry Matter (%) Values of the Bread Samples^a^

	variation/correlation			
samples		Y	M	Y × M
pH				
CC	4.94 ± 0.01^a^	*P* < 0.001	*P* < 0.001	*P* = 0.275
C1	4.80 ± 0.06^bc^	*r* = −0.740**	*r* = −0.249	*r* = n/a
C2	4.59 ± 0.02^de^			
C3	4.87 ± 0.09^ab^			
IC	4.69 ± 0.04^cd^			
I1	4.41 ± 0.03^f^			
I2	4.35 ± 0.01^f^			
I3	4.54 ± 0.10^e^			
*a*_w_				
CC	0.970 ± 0.01^a^	*P* = 0.003	*P* = 0.004	*P* = 0.842
C1	0.967 ± 0.01^bc^	*r* = −0.523*	*r* = −0.135	*r* = n/a
C2	0.965 ± 0.01^cd^			
C3	0.969 ± 0.01^ab^			
IC	0.967 ± 0.01^bc^			
I1	0.965 ± 0.01^cd^			
I2	0.963 ± 0.01^d^			
I3	0.967 ± 0.01^bc^			
Dry Matter (%)				
CC	71.11 ± 0.32^a^	*P* = 0.825	*P* = 0.906	*P* = 0.6022
C1	71.08 ± 0.35^a^	*r* = −0.070	*r* = −0.123	*r* = n/a
C2	70.91 ± 0.37^a^			
C3	70.65 ± 0.46^a^			
IC	70.71 ± 0.52^a^			
I1	70.90 ± 0.19^a^			
I2	71.01 ± 0.31^a^			
I3	70.96 ± 0.44^a^			

a Y: yeast, M: magnetic field intensity, CC: commercial control, C1: commercial culture exposed to 180 μT, C2: commercial culture exposed to 240 μT, C3: commercial culture exposed to 300 μT, IC: isolated control, I1: isolated culture exposed to 180 μT, I2: isolated culture exposed to 240 μT, I3: isolated culture exposed to 300 μT, a–f (↓): values with the different lowercase letters in the same column for each analysis differ significantly (*P* < 0.05). ±: standard deviation. **: correlation is significant at the 0.01 level (2-tailed), *: correlation is significant at the 0.05 level (2-tailed), n/a: nonapplicable.

Studies have shown that exposure to magnetic fields can modulate the activities of key metabolic enzymes in *S. cerevisiae*, affecting the overall cellular energy metabolism and growth dynamics of this yeast species.^10^ The change in cellular energy metabolism affected the fermentation capacity of yeast. The increase in fermentation efficiency also affected the decrease in pH value.

The *a*_w_ values of the samples decreased depending on the magnetic field application intensity (p < 0.05). Although there was a statistical difference, magnetic field application did not highly affect the water activity values of the samples made using isolated and commercial cultures.

Water activity (*a*_w_) is a critical factor of microbial growth, as it delineates the accessibility of free, unbound water essential for microbial metabolism and reproduction. In microbiology, it is commonly acknowledged that water activity, rather than water quantity, is the principal factor influencing microbial growth potential in many media, including food products.^29^ *a*_w_ is a key parameter in the making and storage of bread. It is a measure of water available for microbial growth and chemical reactions and can significantly affect the shelf life, texture and overall quality of bread.^30–33^ The metabolism of yeast is another factor in fermentation that might influence the water activity of bread. Yeasts can affect bread’s *a*_w_ by producing carbon dioxide and other metabolites that enhance the dough’s leavening and texture. The impact of yeast mainly pertains to the fermentation products derived from the transformation of carbohydrates in aerobic or anaerobic conditions. The production of ethyl alcohol and CO_2_ in yeast fermentation depends mainly on glycometabolism, the primary source of carbon elements required for cell synthesis of various substances.^33^ The amount and variety of metabolites produced affect the *a*_w_ value of the final product.

The dry matter values of the samples ranged from 70.65 to 71.11 (Table 1). The highest dry matter value was found in the control sample (71.11%), while the lowest was found in samples produced with commercial yeast exposed to a magnetic field intensity of 300 μT. Although the dry matter values of the bread produced differed depending on the magnetic field intensity applied to the samples and the type of yeast used in production, this difference was found to be statistically insignificant (*p* > 0.05).

Leavening and pore diameter values of the bread samples are shown in Table 2. Yeast strain interaction was very highly significant (p < 0.001) on both leavening and pore diameter values. Magnetic field intensity interaction was very highly significant (p < 0.001) on pore diameter values, where it was significant (p < 0.05) on leavening values.

**Table 2:** Leavening and Pore Diameter Values of the Bread Samples^a^

	variation/correlation			
samples		Y	M	Y × M
Leavening (cm)				
CC	7.96 ± 0.28^de^	*P* = 0.03	*P* < 0.001	*P* = 0.259
C1	8.51 ± 0.21^cd^	*r* = 0.368	*r* = 0.010	*r* = n/a
C2	10.03 ± 0.61^ab^			
C3	7.19 ± 0.44^e^			
IC	8.54 ± 0.31^cd^			
I1	9.27 ± 0.35^bc^			
I2	10.30 ± 0.20^a^			
I3	8.52 ± 0.17^cd^			
Pore Diameter (mm)				
CC	3.38 ± 0.01^d^	*P* < 0.001	*P* < 0.001	*P* = 0.159
C1	4.35 ± 0.23^b^	*r* = 0.413	*r* = 0.169	*r* = n/a
C2	5.01 ± 0.19^a^			
C3	3.79 ± 0.26^c^			
IC	4.21 ± 0.15^b^			
I1	4.98 ± 0.08^a^			
I2	5.35 ± 0.15^a^			
I3	4.10 ± 0.05^bc^			
Volume (cm^3^)				
CC	283.33 ± 2.80^d^	*P* = 0.314	*P* < 0.001	*P* = 0.015
C1	314.15 ± 4.32^b^	*r* = −0.058	*r* = 0.041	*r* = n/a
C2	331.28 ± 0.53^a^			
C3	270.44 ± 9.46^e^			
IC	280.48 ± 1.25^de^			
I1	299.07 ± 2.20^c^			
I2	325.59 ± 2.90^a^			
I3	284.08 ± 6.43^d^			
Specific Volume (cm^3^ g^–1^)				
CC	5.13 ± 0.25^d^	*P* = 0.304	*P* < 0.001	*P* = 0.098
C1	6.12 ± 0.28^ab^	*r* = 0.119	*r* = 0.484	*r* = n/a
C2	6.27 ± 0.22^a^			
C3	5.34 ± 0.25^cd^			
IC	5.55 ± 0.05^cd^			
I1	5.76 ± 0.02^bc^			
I2	6.41 ± 0.12^a^			
I3	5.58 ± 0.19^cd^			

a Y: yeast, M: magnetic field intensity, CC: commercial control, C1: commercial culture exposed to 180 μT, C2: commercial culture exposed to 240 μT, C3: commercial culture exposed to 300 μT, IC: isolated control, I1: isolated culture exposed to 180 μT, I2: isolated culture exposed to 240 μT, I3: isolated culture exposed to 300 μT, a–e (↓): values with the different lowercase letters in the same column for each analysis differ significantly (*P* < 0.05). ±: standard deviation. n/a: nonapplicable.

The leavening values of the bread samples increased in parallel with the magnetic field intensity (p < 0.05). This increase decreased at magnetic field intensities higher than 240 μT. In addition, between two different yeast strains, samples isolated from sourdough samples showed more leavening after magnetic field application compared to commercial cultures. The lowest leavening value among the samples was 7.96 cm in the commercial culture control sample (CC). In contrast, the highest leavening value was 10.30 cm in the I2 sample exposed to a 240 μT magnetic field strength (Table 2).

Similarly, the pore diameters of the bread samples increased up to 240 μT magnetic field intensity, but the pore diameter decreased when the intensity exceeded this value (300 μT). Among the samples, the largest pore diameter was measured in the I2 (isolated yeast exposed to 240 μT magnetic field intensity) sample at 5.35 mm, and the lowest pore diameter was measured in the CC (commercial culture control) sample at 3.38 mm (Table 2).

Dough leavening value and pore diameter are closely related to the CO_2_ produced during fermentation. CO_2_ produced during bread fermentation plays a critical role in the fermentation and texture of the final product. CO_2_ is trapped within the gluten network of the dough and forms air pockets that give the bread its light and airy texture. The production rate of CO_2_, the mechanical characteristics of the dough, and the influence of gravity dictate the final shape and volume of the cooked bread. Yeast metabolizes carbohydrates during bread production, generating CO_2_ and ethanol, crucial for leavening and taste enhancement.^34,35^ The application of magnetic fields is recognized for its role in regulating the activities of fundamental metabolic enzymes, including those associated with glycolysis and respiration.^36^

Magnetic field intensity interaction was very highly significant (p < 0.001) on both volume and specific volume values of bread samples (Table 2). The volume and specific volume of the samples increased with the application of up to 240 μT (p < 0.05) but decreased at higher intensities. The increase in bread volume correlates with the CO_2_ gas produced by yeast during fermentation and, to a lesser extent, during baking.^37,38^ Magnetic fields serve as tools that can facilitate the acceleration of microorganism growth.^39^ Consequently, the application of magnetic fields may significantly enhance the fermentation process.^40^ Research indicates that the application of low-dose magnetic fields positively influences the growth of microorganisms.^18^ Conversely, the application of high doses and prolonged exposure to magnetic fields results in a deceleration and potential cessation of this growth.^20^ The application of magnetic fields is thought to enhance CO_2_ production by improving the fermentation capacity of *S. cerevisiae*, which in turn enhances the leavening and pore structure of bread, resulting in increased volume.

The effects of yeast strain and magnetic field intensity on bread quality were clearly observed across all measured parameters. Isolated sourdough yeast consistently produced lower pH values and slightly reduced water activity compared to commercial cultures, indicating higher metabolic activity and responsiveness to magnetic field stimulation. Leavening and pore diameter also reflected this trend: breads made with isolated yeasts exhibited greater dough expansion and larger pores, with maximum leavening (10.30 cm) and pore size (5.35 mm) observed in the I2 sample (isolated yeast at 240 μT), while the commercial culture control (CC) showed the lowest values (7.96 cm and 3.38 mm). Overall, moderate magnetic field intensities (180–240 μT) enhanced fermentation performance and bread structure, particularly in isolated yeast cultures, whereas excessive intensity (300 μT) slightly inhibited these improvements. These results highlight that strain selection and optimal magnetic field application are key factors for maximizing bread fermentation efficiency and quality.

Textural properties of dough samples are given in Table 3. Magnetic field intensity interaction was very highly significant (p < 0.001) on adhesiveness, springiness, gumminess, and resilience values. Similarly, yeast strain interaction was very highly significant (p < 0.001) on adhesiveness and gumminess values. Furthermore, it was revealed that yeast strain interaction had a highly negative correlative effect on the hardness value (Table 3).

**Table 3:** TPA Results of the Dough Samples^a^

	variation/correlation			
samples		Y	M	Y × M
Hardness (N)				
CC	205.27 ± 3.71^ab^	*P* = 0.007	*P* = 0.012	*P* = 0.702
C1	202.98 ± 1.88^ab^	*r* = −0.539*	*r* = −0.196	*r* = n/a
C2	197.17 ± 1.45^bc^			
C3	206.80 ± 3.45^a^			
IC	201.43 ± 2.99^ab^			
I1	198.62 ± 3.87^ab^			
I2	189.22 ± 5.67^c^			
I3	198.27 ± 2.91^ab^			
Cohesiveness				
CC	0.725 ± 0.01^ab^	*P* = 0.422	*P* = 0.053	*P* = 0.381
C1	0.665 ± 0.03^b^	*r* = 0.172	*r* = 0.165	*r* = n/a
C2	0.620 ± 0.01^b^			
C3	0.703 ± 0.01^ab^			
IC	0.686 ± 0.01^ab^			
I1	0.671 ± 0.01^ab^			
I2	0.650 ± 0.01^b^			
I3	0.788 ± 0.13^a^			
Adhesiveness (g·s)				
CC	–0.165 ± 0.06^a^	*P* < 0.001	*P* < 0.001	*P* = 0.059
C1	–0.385 ± 0.08^b^	*r* = −0.448	*r* = 0.072	*r* = n/a
C2	–0.485 ± 0.04^b^			
C3	–0.145 ± 0.05^a^			
IC	–0.420 ± 0.08^b^			
I1	–0.480 ± 0.06^b^			
I2	–0.780 ± 0.04^c^			
I3	–0.220 ± 0.01^a^			
Springiness				
CC	0.858 ± 0.01^a^	*P* = 0.298	*P* < 0.001	*P* < 0.001
C1	0.830 ± 0.01^bc^	*r* = −0.070	*r* = −0.314	*r* = n/a
C2	0.739 ± 0.01^e^			
C3	0.838 ± 0.01^ab^			
IC	0.825 ± 0.01^bc^			
I1	0.811 ± 0.01^cd^			
I2	0.794 ± 0.01^d^			
I3	0.817 ± 0.01^c^			
Gumminess				
CC	148.90 ± 0.52^a^	*P* < 0.001	*P* < 0.001	*P* < 0.001
C1	135.11 ± 7.71^abc^	*r* = −0.478	*r* = −0.008	*r* = n/a
C2	122.24 ± 0.28^c^			
C3	145.46 ± 0.24^ab^			
IC	138.19 ± 3.19^abc^			
I1	133.29 ± 4.56^abc^			
I2	123.10 ± 4.36^bc^			
I3	156.05 ± 23.49^a^			
Chewiness				
CC	127.76 ± 1.28^a^	*P* = 0.720	*P* = 0.011	*P* = 0.185
C1	112.17 ± 4.97^abc^	*r* = −0.047	*r* = −0.039	*r* = n/a
C2	90.33 ± 0.73^d^			
C3	121.97 ± 0.93^ab^			
IC	113.99 ± 1.26^abc^			
I1	108.08 ± 2.76^bc^			
I2	97.71 ± 1.89^cd^			
I3	127.41 ± 18.09^a^			
Resilience				
CC	0.310 ± 0.01^a^	*P* = 0.011	*P* < 0.001	*P* = 0.449
C1	0.281 ± 0.01^c^	*r* = −0.243	*r* = −0.271	*r* = n/a
C2	0.247 ± 0.01^d^			
C3	0.302 ± 0.01^ab^			
IC	0.292 ± 0.01^bc^			
I1	0.277 ± 0.01^c^			
I2	0.239 ± 0.01^d^			
I3	0.286 ± 0.02^bc^			

a Y: yeast, M: magnetic field intensity, CC: commercial control, C1: commercial culture exposed to 180 μT, C2: commercial culture exposed to 240 μT, C3: commercial culture exposed to 300 μT, IC: isolated control, I1: isolated culture exposed to 180 μT, I2: isolated culture exposed to 240 μT, I3: isolated culture exposed to 300 μT, a–e (↓): values with the different lowercase letters in the same column for each analysis differ significantly (*P* < 0.05). ±: standard deviation. *: correlation is significant at the 0.05 level (2-tailed), n/a: nonapplicable.

Textural values of all dough samples showed a decrease in the samples exposed to magnetic field application (p < 0.05). The degree of this decrease increased in the samples exposed to magnetic field applications above 240 μT intensity. In addition, of the two different yeast species used, the reduction of isolated yeast samples was much more than that of commercial cultures (*p* < 0.05; Table 3). It was determined that the samples showing the highest decrease in all texture values were the samples exposed to a 240 μT magnetic field intensity. On the other hand, the highest values occurred in commercial culture control samples.

The fermentation process conducted by *S. cerevisiae* significantly affects the texture of bread dough. Yeast generates different metabolites during fermentation that can considerably influence the rheological characteristics of the dough. A key factor is the generation of carbon dioxide, which facilitates fermentation and the expansion of the dough, yielding a softer and more aerated texture.^33,41^ Additionally, the release of organic acids, including lactic and acetic acid, may influence the extensibility and viscoelasticity of the dough, thereby impacting the final texture of the bread.^42^ Applying a magnetic field enhanced the production of CO_2_ and other metabolites by altering the fermentation capacity of yeast. This is thought to improve the textural properties of the dough samples.

Textural properties of bread samples are given in Table 4. Yeast strain interaction was very highly significant (p < 0.001) on TPA values of gumminess and chewiness, whereas magnetic field intensity interaction was very highly significant (p < 0.001) on gumminess, chewiness, and resilience. Furthermore, it was revealed that yeast strain interaction had a highly negative correlative effect on the hardness value (Table 4).

**Table 4:** TPA Results of the Bread Samples^a^

	variation/correlation			
samples		Y	M	Y × M
Hardness (N)				
CC	205.27 ± 3.72^ab^	*P* = 0.070	*P* = 0.12	*P* = 0.07
C1	202.98 ± 1.89^ab^	*r* = −0.509*	*r* = −0.196	*r* = n/a
C2	197.17 ± 1.35^bc^			
C3	206.80 ± 3.46^a^			
IC	201.43 ± 2.99^ab^			
I1	198.62 ± 3.87^ab^			
I2	189.22 ± 5.68^c^			
I3	198.27 ± 2.92^ab^			
Cohesiveness				
CC	0.895 ± 0.01^ab^	*P* = 0.002	*P* < 0.001	*P* = 0.160
C1	0.865 ± 0.01^b^	*r* = −0.106	*r* = 0.314	*r* = n/a
C2	0.785 ± 0.02^c^			
C3	0.930 ± 0.03^a^			
IC	0.875 ± 0.01^b^			
I1	0.780 ± 0.01^c^			
I2	0.750 ± 0.01^c^			
I3	0.900 ± 0.03^ab^			
Adhesiveness (g·s)				
CC	–0.85 ± 0.09^a^	*P* = 0.351	*P* = 0.021	*P* = 0.970
C1	–0.91 ± 0.14^ab^	*r* = −0.192	*r* = −0.115	*r* = n/a
C2	–2.80 ± 0.38^bc^			
C3	–0.69 ± 0.04^a^			
IC	–1.46 ± 0.86^abc^			
I1	–1.85 ± 0.71^abc^			
I2	–3.94 ± 1.81^c^			
I3	–1.02 ± 0.09^abc^			
Springiness				
CC	1.01 ± 0.02^bc^	*P* = 0.395	*P* < 0.001	*P* = 0.947
C1	0.98 ± 0.01^cd^	*r* = −0.330	*r* = 0.062	*r* = n/a
C2	0.96 ± 0.01^d^			
C3	1.04 ± 0.01^a^			
IC	0.99 ± 0.01^c^			
I1	0.98 ± 0.01^cd^			
I2	0.96 ± 0.01^d^			
I3	1.03 ± 0.01^ab^			
Gumminess				
CC	525.37 ± 1.07^b^	*P* < 0.001	*P* < 0.001	*P* < 0.001
C1	476.99 ± 5.98^c^	*r* = −0.478	*r* = −0.008	*r* = n/a
C2	384.95 ± 13.94^de^			
C3	597.94 ± 9.03^a^			
IC	483.67 ± 4.85^c^			
I1	399.75 ± 2.37^d^			
I2	359.70 ± 4.99^e^			
I3	458.47 ± 24.47^c^			
Chewiness				
CC	527.99 ± 7.12^b^	*P* < 0.001	*P* < 0.001	*P* < 0.001
C1	469.86 ± 6.54^c^	*r* = −0.439	*r* = 0.070	*r* = n/a
C2	369.65 ± 13.32^de^			
C3	621.79 ± 0.66^a^			
IC	481.27 ± 5.83^c^			
I1	391.73 ± 3.34^d^			
I2	345.28 ± 0.30^e^			
I3	472.05 ± 18.70^c^			
Resilience				
CC	0.43 ± 0.01^a^	*P* = 0.017	*P* < 0.001	*P* = 0.401
C1	0.41 ± 0.01^ab^	*r* = −0.330	*r* = −0.029	*r* = n/a
C2	0.38 ± 0.01^cd^			
C3	0.43 ± 0.02^a^			
IC	0.41 ± 0.01^ab^			
I1	0.38 ± 0.01^cd^			
I2	0.36 ± 0.01^d^			
I3	0.43 ± 0.01^a^			

a Y: yeast, M: magnetic field intensity, CC: commercial control, C1: commercial culture exposed to 180 μT, C2: commercial culture exposed to 240 μT, C3: commercial culture exposed to 300 μT, IC: isolated control, I1: isolated culture exposed to 180 μT, I2: isolated culture exposed to 240 μT, I3: isolated culture exposed to 300 μT, a–e (↓): values with the different lowercase letters in the same column for each analysis differ significantly (*P* < 0.05). ±: standard deviation. *: correlation is significant at the 0.05 level (2-tailed), n/a: nonapplicable.

Except for the adhesiveness value, all other TPA values of bread samples decreased in bread made with cultures exposed to magnetic field applications up to 240 μT intensity but increased in bread with cultures exposed to magnetic field applications at 300 μT intensity (p < 0.05). The value of the decrease was higher in isolated culture samples (*p* < 0.05; Table 4). In parallel with the dough TPA values, the samples with the highest decrease in bread TPA values were the samples made with isolated cultures exposed to 240 μT magnetic field applications.

Organic acid values of the bread samples are shown in Table 5. Yeast strain and magnetic field intensity interactions were very highly significant (p < 0.001) on lactic, oxalic and malic acid values of bread samples. In addition, magnetic field intensity interaction had a highly negative correlative effect on the lactic acid value and yeast strain interaction had a very highly negative correlative effect on the oxalic acid value, a very highly positive correlative effect on the malic acid value and a highly negative correlative effect on the ascorbic acid value (Table 5).

**Table 5:** Organic Acid Contents of the Bread Samples (mg/kg)^a^

	variation/correlation			
samples		Y	M	Y × M
Lactic Acid				
CC	157.50 ± 6.36^g^	*P* < 0.001	*P* < 0.001	*P* < 0.001
C1	172.50 ± 6.36^f^	*r* = −0.246	*r* = −0.770**	*r* = n/a
C2	208.50 ± 3.53^d^			
C3	175.00 ± 8.48^f^			
IC	195.50 ± 3.53^e^			
I1	262.50 ± 4.94^b^			
I2	291.00 ± 4.24^a^			
I3	238.00 ± 5.65^c^			
Ascorbic Acid				
CC	0.726 ± 0.05^a^	*P* = 0.664	*P* = 0.084	*P* = 0.974
C1	0.737 ± 0.05^a^	*r* = −0.533*	*r* = 0.188	*r* = n/a
C2	0.754 ± 0.05^a^			
C3	0.739 ± 0.05^a^			
IC	0760 ± 0.07^a^			
I1	0.785 ± 0.03^a^			
I2	0.817 ± 0.02^a^			
I3	0.780 ± 0.03^a^			
Citric Acid				
CC	3.036 ± 0.03^c^	*P* = 0.096	*P* = 0.051	*P* = 0.184
C1	3.085 ± 0.02^bc^	*r* = −0.432	*r* = −0.266	*r* = n/a
C2	3.383 ± 0.29^a^			
C3	3.026 ± 0.03^c^			
IC	3.190 ± 0.03^abc^			
I1	3.243 ± 0.11^abc^			
I2	3.291 ± 0.02^abc^			
I3	3.322 ± 0.01^ab^			
Oxalic Acid				
CC	0.090 ± 0.02^c^	*P* < 0.001	*P* < 0.001	*P* = 0.002
C1	0.121 ± 0.02^bc^	*r* = −0.835**	*r* = 0.328	*r* = n/a
C2	0.122 ± 0.01^bc^			
C3	0.117 ± 0.01^bc^			
IC	0.146 ± 0.02^b^			
I1	0.286 ± 0.03^a^			
I2	0.296 ± 0.01^a^			
I3	0.277 ± 0.01^a^			
Tartaric Acid				
CC	0.017 ± 0.01^a^	*P* = 0.310	*P* = 0.574	*P* = 0.474
C1	0.055 ± 0.02^a^	*r* = −0.150	*r* = 0.141	*r* = n/a
C2	0.416 ± 0.50^a^			
C3	0.043 ± 0.02^a^			
IC	0.061 ± 0.01^a^			
I1	0.097 ± 0.02^a^			
I2	0.110 ± 0.01^a^			
I3	0.056 ± 0.11^a^			
Malic Acid				
CC	1.935 ± 0.04^e^	*P* < 0.001	*P* < 0.001	*P* < 0.001
C1	1.980 ± 0.07^e^	*r* = 0.836**	*r* = 0.334	*r* = n/a
C2	2.155 ± 0.02^de^			
C3	2.255 ± 0.11^cd^			
IC	2.415 ± 0.09^c^			
I1	3.700 ± 0.08^b^			
I2	3.955 ± 0.09^a^			
I3	3.520 ± 0.22^b^			

a Y: yeast, M: magnetic field intensity, CC: commercial control, C1: commercial culture exposed to 180 μT, C2: commercial culture exposed to 240 μT, C3: commercial culture exposed to 300 μT, IC: isolated control, I1: isolated culture exposed to 180 μT, I2: isolated culture exposed to 240 μT, I3: isolated culture exposed to 300 μT, a–g (↓): values with the different lowercase letters in the same column for each analysis differ significantly (*P* < 0.05). ±: standard deviation. **: correlation is significant at the 0.01 level (2-tailed), *: correlation is significant at the 0.05 level (2-tailed), n/a: nonapplicable.

Applying a magnetic field caused an increase in the levels of organic acids in the bread samples (p < 0.05). The increase was higher in samples made with cultures subjected to magnetic field intensity of up to 240 μT, whereas it was less significant in the bread made with cultures subjected to higher magnetic field intensity of 300 μT (*p* < 0.05; Table 5). The type of culture used also had an effect on the increase in organic acid values (p < 0.05). The increase in organic acid values in bread made with isolated cultures was higher compared to commercial cultures.

Compared to the control samples, magnetic field application increased the organic acid amounts of the samples in general (p < 0.05). Among the organic acids, the highest increase was found in lactic, malic and citric acids. Similar to our other results, the increase in organic acid values of bread made with cultures isolated from sourdough samples and exposed to a magnetic field intensity of 240 μT was higher than those made with commercial cultures (p < 0.05).

The fermentation carried out by *S. cerevisiae* not only produces carbon dioxide to leaven the dough but also various organic acids that contribute to the flavor profile of the final bread product.^34^ The fermentation of *S. cerevisiae* significantly influences the organic acid profile of bread, resulting in the production of lactic and acetic acids. These acids are byproducts of yeast glycometabolism, resulting from the conversion of sugars under both aerobic and anaerobic conditions, which leads to the accumulation of these organic acids.^33^ The relative concentrations of lactic and acetic acids in bread can significantly affect its overall taste, with lactic acid imparting a milder, more subtle flavor and acetic acid adding a zingier, bolder flavor.^3^

The quantity and diversity of organic acids produced during fermentation depend on the yeast type and fermentation conditions. Research has explored the use of magnetic fields as a means to modulate the metabolic processes of diverse microorganisms, including *S. cerevisiae*, a yeast of significant industrial relevance.^42^ Magnetic fields have demonstrated an impact on cellular processes in living organisms, including growth, pigment production, and the synthesis of osmolytes, nitric oxide, and hydrogen sulfide.^43^ The application of magnetic fields may be utilized to regulate the metabolic activities of *S. cerevisiae*, including their ability to produce organic acids. While the exact mechanisms behind these effects remain incomplete, numerous studies indicate that magnetic fields may influence cellular processes, including growth, membrane integrity, and the activity of cellular compounds and organelles.^6,42–44^

The textural and organic acid profiles of both dough and bread samples were significantly influenced by yeast strain and magnetic field intensity. Textural analysis showed that dough and bread samples made with isolated sourdough yeast were more responsive to magnetic field applications than those made with commercial cultures. Specifically, TPA parameters such as hardness, gumminess, chewiness, and resilience decreased under moderate magnetic field intensities (up to 240 μT), with the most pronounced reduction observed in isolated yeast samples. Excessive intensity (300 μT) partially reversed these improvements, particularly in commercial cultures. This suggests that moderate magnetic fields enhance dough softness, elasticity, and overall viscoelastic properties, likely through increased CO_2_ and metabolite production during fermentation.

Similarly, the levels of organic acids in bread were markedly affected by yeast type and magnetic field intensity. Application of moderate magnetic fields (up to 240 μT) increased the concentrations of lactic, malic, and citric acids, with isolated yeast cultures showing higher organic acid accumulation than commercial cultures. At higher field intensity (300 μT), the increase was less pronounced, indicating that excessive magnetic exposure may suppress metabolite synthesis. These results suggest that moderate magnetic fields can enhance both textural quality and the biochemical profile of bread, improving dough rheology, fermentation efficiency, and flavor development, particularly when using isolated sourdough yeast.

## Conclusion

This study aimed to examine the biological valence, qualitative characteristics, and dough fermentation efficiency of bread samples made with *S. cerevisiae*, also referred to as bakery yeast, when exposed to magnetic field stress.

The pH, *a*_w_, leavening, pore diameter, TPA and organic acid values of dough made with yeast cultures exposed to magnetic field intensity up to 240 μT and bread samples made from these doughs increased compared to control samples. However, this positive effect showed a negative change in dough and bread made with cultures exposed to magnetic field intensities above 240 μT.

Bread, rich in carbohydrates, is widely consumed as a staple food globally. The fermentation stage is crucial in determining the quality and nutritional value of bread. *S. cerevisiae*, commonly referred to as bread yeast, influences bread’s technological and sensory characteristics through the production of metabolites such as CO_2_ and ethanol during fermentation. The metabolite activities of *S. cerevisiae* can be enhanced through exposure to a magnetic field. This study utilized *S. cerevisiae*, which was exposed to varying intensities of magnetic fields in bread making. The physicochemical and textural properties of the bread samples that were made were analyzed.

The application of a magnetic field was found to improve the fermentation capacity of *S. cerevisiae*. In this context, the quantity of CO_2_ generated in the bread increased, contributing to an improved leavening rate and pore diameters of the bread. Simultaneously, there was an increase in the concentration of organic acids in the bread samples made with yeast subjected to a magnetic field. As a result, the pH levels in these samples exhibited a more significant decrease. The analysis results revealed improvements in the textural properties of both dough and bread samples. The changes in textural properties are associated with the quantity and diversity of metabolites produced by *S. cerevisiae*, mainly CO_2_ and organic acids.

The study’s results indicate that using a magnetic field in *S. cerevisiae* culture markedly improved bread’s techno-functional capabilities and nutritional quality. Future research should examine the potential of magnetic field application to enhance the capacity of *S. cerevisiae* to produce various metabolites, particularly organic acids, in industrial settings.
